# (*Z*)-1-(2-Chloro­phen­yl)-3-methyl-4-[2-(4-nitro­phen­yl)hydrazin-1-yl­idene]-1*H*-pyrazol-5(4*H*)-one

**DOI:** 10.1107/S1600536812029790

**Published:** 2012-07-07

**Authors:** Carlos Bustos, Andrés Escobar-Fuentealba, Luis Alvarez-Thon, Rodrigo Faundez-Gutierrez, Maria Teresa Garland

**Affiliations:** a Instituto de Ciencias Químicas, Universidad Austral de Chile, Avenida Los Robles s/n, Campus Isla Teja, Casilla 567, Valdivia, Chile; b Departamento de Ciencias Físicas, Universidad Andres Bello, Avenida República 220, Santiago de Chile, Chile; c Laboratorio de Cristalografía, Departamento de Física, Facultad de Ciencias Físicas y Matemáticas, Universidad de Chile, Avenida Blanco Encalada 2008, Santiago de Chile, Chile

## Abstract

There are two independent mol­ecules, *A* and *B*, in the asymmetric unit of the title compound, C_16_H_12_ClN_5_O_3_. The relative orientations of the chloro­phenyl ring with respect to the pyrazole ring in the two crystallographically independent mol­ecules are different, and their corresponding dihedral angles are −53.3 (2) and 114.09 (18)° in mol­ecules *A* and *B*, respectively. There are two strong intramolecular N—H⋯O hydrogen bonds, and two weak intramolecular C—H⋯O and C—H⋯Cl hydrogen bonds. The crystal packing is constructed by weak C—H⋯O and N—H⋯O inter­actions, and two π–π stacking inter­actions [centroid–centroid distances = 3.7894 (9) and 3.5719 (10) Å], forming a mol­ecular ladder along the *a* axis.

## Related literature

For synthesis and related literature, see: Abdel-Aziz *et al.* (2009 ▶); Bustos *et al.* (2006 ▶, 2007 ▶, 2009 ▶, 2012 ▶). For the biological activity of this class of compounds, see: Castagnolo *et al.* (2009 ▶); Chauhan *et al.* (1993 ▶); El-Hawash *et al.* (2006 ▶); Gunasekaran *et al.* (2011 ▶); Himly *et al.* (2003 ▶); Jolly *et al.* (1991 ▶); Kalluraya *et al.* (2007 ▶); Kawai *et al.* (1997 ▶); Moreau *et al.* (2008 ▶); Pasha *et al.* (2009 ▶); Radi *et al.* (2009 ▶); Singh (1991 ▶); Wu *et al.* (2002 ▶).
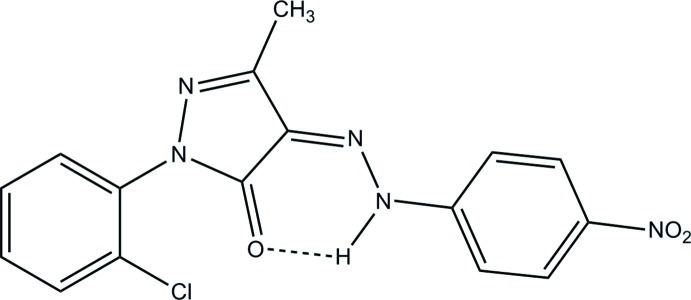


## Experimental

### 

#### Crystal data

C_16_H_12_ClN_5_O_3_
*M* *_r_* = 357.76
Triclinic, 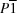

*a* = 7.2715 (6) Å
*b* = 14.7757 (12) Å
*c* = 15.7609 (12) Å
α = 75.408 (1)°
β = 86.943 (1)°
γ = 79.774 (1)°
*V* = 1612.7 (2) Å^3^
*Z* = 4
Mo *K*α radiation
μ = 0.26 mm^−1^
*T* = 150 K
0.24 × 0.22 × 0.10 mm

#### Data collection

Bruker D8 Discover diffractometer with SMART CCD area-detector
13022 measured reflections
6469 independent reflections
4430 reflections with *I* > 2σ(*I*)
*R*_int_ = 0.024

#### Refinement

*R*[*F* ^2^ > 2σ(*F* ^2^)] = 0.039
*wR*(*F* ^2^) = 0.097
*S* = 0.91
6469 reflections
461 parameters
H atoms treated by a mixture of independent and constrained refinement
Δρ_max_ = 0.44 e Å^−3^
Δρ_min_ = −0.50 e Å^−3^

### 

Data collection: *SMART* (Bruker, 2001 ▶); cell refinement: *SAINT* (Bruker, 2000 ▶); data reduction: *SAINT*; program(s) used to solve structure: *SHELXS97* (Sheldrick, 2008 ▶); program(s) used to refine structure: *SHELXL97* (Sheldrick, 2008 ▶); molecular graphics: *XP* in *SHELXTL-PC* (Sheldrick, 2008 ▶); software used to prepare material for publication: *PLATON* (Spek, 2009 ▶) and *Mercury* (Macrae *et al.*, 2008 ▶).

## Supplementary Material

Crystal structure: contains datablock(s) global, I. DOI: 10.1107/S1600536812029790/ds2200sup1.cif

Structure factors: contains datablock(s) I. DOI: 10.1107/S1600536812029790/ds2200Isup2.hkl

Supplementary material file. DOI: 10.1107/S1600536812029790/ds2200Isup3.cml

Additional supplementary materials:  crystallographic information; 3D view; checkCIF report

## Figures and Tables

**Table 1:** Hydrogen-bond geometry (Å, °)

*D*—H⋯*A*	*D*—H	H⋯*A*	*D*⋯*A*	*D*—H⋯*A*
N4—H7⋯O1	0.910 (19)	2.084 (18)	2.777 (2)	132.1 (14)
N9—H8⋯O4	0.863 (19)	2.222 (18)	2.8647 (19)	131.2 (16)
C28—H28⋯O2	0.95	2.57	3.265 (2)	130
C15—H15⋯Cl2	0.95	2.93	3.4770 (18)	118
N9—H8⋯O2	0.86 (2)	2.654 (18)	3.266 (2)	129.0 (13)
N9—H8⋯O1^i^	0.863 (19)	2.478 (17)	3.094 (2)	128.9 (14)
C4—H4⋯O5^ii^	0.95	2.37	3.202 (3)	146
C10—H10*B*⋯O3^iii^	0.98	2.52	3.392 (3)	148
C10—H10*C*⋯O6^iv^	0.98	2.43	3.175 (3)	133
C32—H32⋯Cl2^v^	0.95	2.80	3.6898 (18)	157

Symmetry codes: (i) 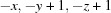; (ii) 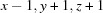; (iii) 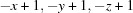; (iv) 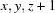; (v) 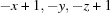.

**Fig. 1.**
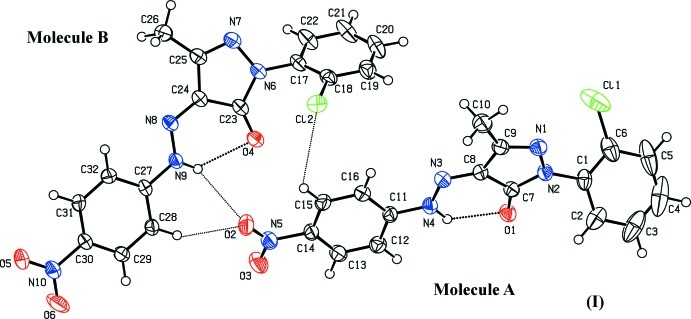
View of the title compound with the atom numbering scheme. Displacement ellipsoids for non-H atoms are drawn at the 50% probability level. The strong intramolecular hydrogen bonds (N9—H8···O4 and N4—H7···O1) are depicted with dashed lines. The weak intramolecular contacts (N9—H8···O2, C15—H15···Cl2 and C28—H28···O2) are depicted with dotted lines.

**Fig. 2.**
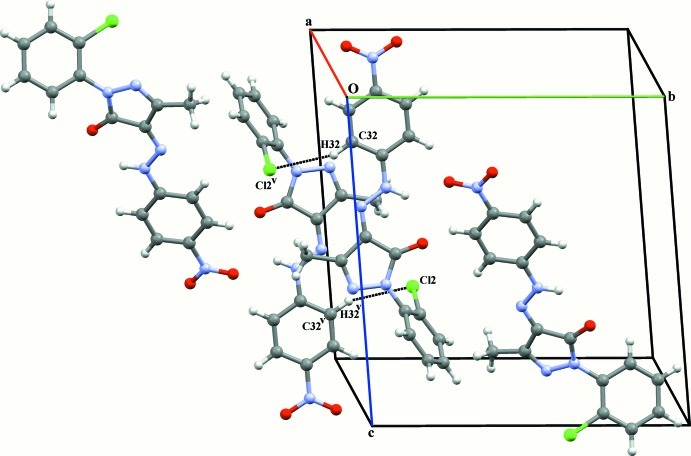
Part of the crystal packing showing the formation of an invertion-related dimer via C12—H32···Cl2v weak contacts (dashed lines). [Symmetry code: (v) -x + 1, -y, -z + 1].

**Fig. 3.**
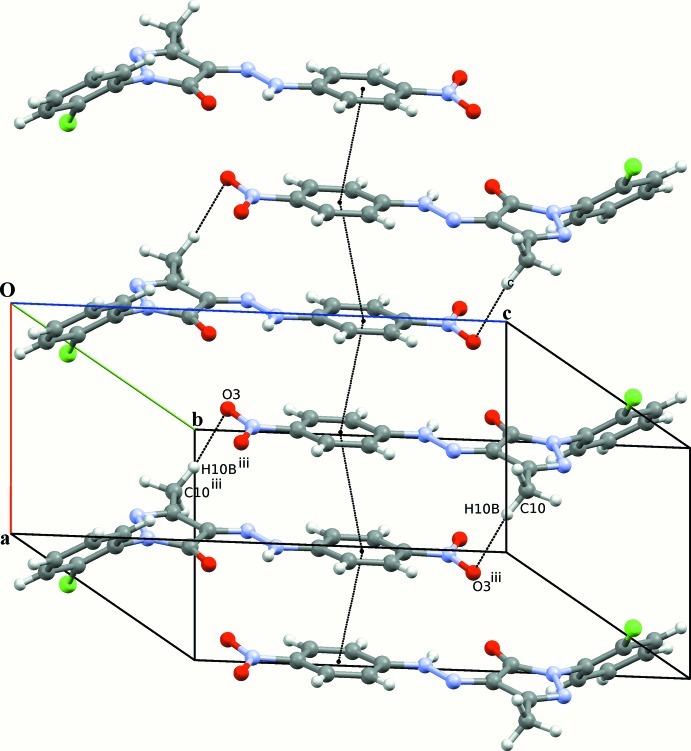
Part of the crystal packing showing the formation of a dimer via C10—H10B···O3iii weak contacts (dashed lines). In addition, there are two π–π stacking interactions (Cg···Cgi and Cg···Cgiii) that form a molecular ladder along the a direction, where Cg is the centroid of the C11—C16 ring. [Symmetry codes: (i) -x, -y + 1, -z + 1; (iii) -x + 1, -y + 1, -z + 1].

## supplementary crystallographic information

### Comment

In previous papers, we have informed preliminary results about the synthesis of a large library of pyrazoles by reaction of β-diketohydrazones with substituted arylhydrazines (Bustos *et al.*, 2009) and three molecular structures of this type of compounds have been reported (Bustos *et al.*, 2006, 2007, 2012). As a consequence of these studies we have found that α-hydrazo-β-ketoesters yield pyrazolones by reaction with substituted arylhydrazines. Now, we present the synthesis and the molecular structure of the title compound, prepared by reaction of (*E*)-ethyl 2-(2-(4-nitrophenyl)hydrazinylidene)-3-oxobutanoate with (2-chlorophenyl)hydrazine.

The title compound shown in Fig. 1, crystallizes with two independent molecules in the asymmetric unit, where the chlorophenyl ring display a different orientation with respect to the pyrazole ring in molecules A and B. The corresponding dihedral angles between the chlorophenyl and pyrazole rings, in molecule A and molecule B are -53.3 (2)° and 114.09 (18)° respectively. In the crystal, two strong intramolecular hydrogen bonds (N4—H7···O1 and N9—H8···O4) are observed (Fig. 1, Table 1). As shown in Fig. 1 and Table 1, there are three additional weak intramolecular contacts that link molecules A and B (N9—H8···O2, C15—H15···Cl2 and C28—H28···O2). The atoms in the C1—C6 ring are slightly disordered due to thermal motion.

The partial packing of (I), shows that the two molecules in the asymmetric unit form inversion dimers *via* a pair of weak C32—H32···Cl2^v^ bonds (Fig. 2, Table 1). There is another pair of weak C10—H10B···O3^iii^ bonds that form a dimer involving molecule A. In addition, there are two π–π stacking interactions, one with *Cg*···*Cg*^i^ distance of 3.6112 (9) Å and the other with *Cg*···*Cg*^iii^ distance of 3.7894 (9) Å, where *Cg* is the centroid of the C11–C16 ring. These interactions form a molecular ladder that runs parallel to the *a* axis (Fig. 3, Table 1). Finally the crystal packing is completed with three weak contacts: C4—H4···O5^ii^, N9—H8···O1^i^ and C10—H10C···O6^iv^ (Table 1). [Symmetry codes: (i) -*x*, -*y* + 1, -*z* + 1; (ii) *x* - 1, *y* + 1, *z* + 1; (iii) -*x* + 1, -*y* + 1, -*z* + 1; (iv) *x*, *y*, *z* + 1; (v) -*x* + 1, -*y*, -*z* + 1].

### Experimental

In a 100 ml round-bottomed flask were added 2.34 g (8.38 mmole) of (*E*)-ethyl 2-(2-(4-nitrophenyl)hydrazinylidene)-3-oxobutanoate, 1.60 g (8.94 mmole) 2-(2-chlorophenyl)hydrazine hydrochloride, 5 ml of glacial acetic acid and 50 ml of ethanol. The reaction mixture was magnetically stirred and heated under reflux during 36 h. Then, after cooling at room temperature, the precipitate was filtrated by suction and dried in a vacuum oven at 45°C during 24 h. Yield 78% of crude product. Single crystals suitable for X-ray studies were obtained by recrystallization from tetrahydrofuran. Melting point: 182–183 °C.

### Refinement

All H atoms were found in difference Fourier maps. The H atoms attached to the N9 and N4 atoms were refined freely against the difraction data, but all other H atoms were placed in geometrically idealized positions and constrained to ride on their parent atoms, with aromatic C—H = 0.95 Å, methyl C—H = 0.98 Å and *U*_iso_(H) = 1.2*U*_eq_(aromatic C) or *U*_iso_(H) = 1.5*U*_eq_(methyl C).

### Crystal data

**Table:**

C_16_H_12_ClN_5_O_3_	*Z* = 4
*M**_r_* = 357.76	*F*(000) = 736
Triclinic, *P*1	*D*_x_ = 1.474 Mg m^−^^3^
Hall symbol: -P 1	Mo *K*α radiation, λ = 0.71073 Å
*a* = 7.2715 (6) Å	Cell parameters from 999 reflections
*b* = 14.7757 (12) Å	θ = 1.7–26.3°
*c* = 15.7609 (12) Å	µ = 0.26 mm^−^^1^
α = 75.408 (1)°	*T* = 150 K
β = 86.943 (1)°	Polyhedron, orange
γ = 79.774 (1)°	0.24 × 0.22 × 0.10 mm
*V* = 1612.7 (2) Å^3^	

### Data collection

**Table:**

Bruker D8 Discover diffractometer with SMART CCD area-detector	4430 reflections with *I* > 2σ(*I*)
Radiation source: fine-focus sealed tube	*R*_int_ = 0.024
Graphite monochromator	θ_max_ = 26.3°, θ_min_ = 1.7°
φ and ω scans	*h* = −9→9
13022 measured reflections	*k* = −18→18
6469 independent reflections	*l* = −19→18

### Refinement

**Table:**

Refinement on *F*^2^	Primary atom site location: structure-invariant direct methods
Least-squares matrix: full	Secondary atom site location: difference Fourier map
*R*[*F*^2^ > 2σ(*F*^2^)] = 0.039	Hydrogen site location: inferred from neighbouring sites
*wR*(*F*^2^) = 0.097	H atoms treated by a mixture of independent and constrained refinement
*S* = 0.91	*w* = 1/[σ^2^(*F*_o_^2^) + (0.0531*P*)^2^] where *P* = (*F*_o_^2^ + 2*F*_c_^2^)/3
6469 reflections	(Δ/σ)_max_ = 0.001
461 parameters	Δρ_max_ = 0.44 e Å^−^^3^
0 restraints	Δρ_min_ = −0.50 e Å^−^^3^

### Special details

**Table:**

Geometry. Bond distances, angles *etc*. have been calculated using the rounded fractional coordinates. All su's are estimated from the variances of the (full) variance-covariance matrix. The cell e.s.d.'s are taken into account in the estimation of distances, angles and torsion angles
Refinement. Refinement on *F*^2^ for ALL reflections except those flagged by the user for potential systematic errors. Weighted *R*-factors *wR* and all goodnesses of fit *S* are based on *F*^2^, conventional *R*-factors *R* are based on *F*, with *F* set to zero for negative *F*^2^. The observed criterion of *F*^2^ > σ(*F*^2^) is used only for calculating -*R*-factor-obs *etc*. and is not relevant to the choice of reflections for refinement. *R*-factors based on *F*^2^ are statistically about twice as large as those based on *F*, and *R*-factors based on ALL data will be even larger.

### Fractional atomic coordinates and isotropic or equivalent isotropic displacement parameters (Å^2^)

**Table:**

	*x*	*y*	*z*	*U*_iso_*/*U*_eq_	
Cl1	−0.01333 (8)	0.62030 (5)	1.02576 (4)	0.0783 (2)	
O1	0.04690 (16)	0.71458 (8)	0.70638 (7)	0.0356 (4)	
O2	0.39096 (19)	0.34058 (10)	0.33864 (9)	0.0506 (5)	
O3	0.1869 (2)	0.45770 (10)	0.26804 (9)	0.0556 (5)	
N1	0.3064 (2)	0.59306 (10)	0.89571 (9)	0.0348 (5)	
N2	0.17363 (19)	0.66873 (10)	0.84708 (9)	0.0325 (5)	
N3	0.2942 (2)	0.52887 (10)	0.69715 (9)	0.0337 (5)	
N4	0.2057 (2)	0.57477 (11)	0.62260 (9)	0.0325 (5)	
N5	0.2826 (2)	0.41611 (12)	0.33324 (11)	0.0427 (6)	
C1	0.0966 (2)	0.74606 (13)	0.88317 (12)	0.0360 (6)	
C2	0.1016 (3)	0.83741 (13)	0.83373 (15)	0.0472 (7)	
C3	0.0258 (3)	0.91360 (16)	0.8665 (2)	0.0746 (10)	
C4	−0.0534 (4)	0.8998 (2)	0.9486 (3)	0.0919 (14)	
C5	−0.0606 (3)	0.8097 (2)	0.99852 (17)	0.0776 (10)	
C6	0.0128 (3)	0.73186 (16)	0.96534 (13)	0.0509 (7)	
C7	0.1476 (2)	0.65996 (12)	0.76493 (11)	0.0303 (6)	
C8	0.2673 (2)	0.56924 (11)	0.76232 (11)	0.0303 (6)	
C9	0.3580 (2)	0.53480 (12)	0.84638 (11)	0.0334 (6)	
C10	0.4958 (3)	0.44607 (13)	0.87643 (13)	0.0461 (7)	
C11	0.2284 (2)	0.53490 (12)	0.55094 (11)	0.0304 (6)	
C12	0.1493 (2)	0.58808 (12)	0.47130 (11)	0.0345 (6)	
C13	0.1677 (2)	0.54937 (13)	0.39973 (12)	0.0364 (6)	
C14	0.2656 (2)	0.45854 (12)	0.40817 (11)	0.0337 (6)	
C15	0.3460 (2)	0.40554 (12)	0.48640 (11)	0.0347 (6)	
C16	0.3273 (2)	0.44349 (12)	0.55815 (11)	0.0328 (6)	
Cl2	0.38569 (6)	0.19954 (3)	0.65477 (3)	0.0401 (2)	
O4	0.14560 (16)	0.21654 (9)	0.47521 (7)	0.0372 (4)	
O5	0.6349 (2)	0.07315 (9)	−0.02481 (8)	0.0530 (5)	
O6	0.5293 (3)	0.22216 (11)	−0.05986 (9)	0.0832 (7)	
N6	0.1295 (2)	0.08970 (10)	0.59660 (9)	0.0339 (5)	
N7	0.16950 (19)	−0.01115 (10)	0.61679 (9)	0.0338 (5)	
N8	0.31059 (18)	0.04899 (10)	0.39600 (9)	0.0291 (5)	
N9	0.3055 (2)	0.13105 (11)	0.33806 (9)	0.0293 (5)	
N10	0.5588 (2)	0.14595 (11)	−0.00578 (10)	0.0417 (6)	
C17	0.0555 (2)	0.13690 (12)	0.66200 (11)	0.0338 (6)	
C18	0.1585 (2)	0.19249 (12)	0.69232 (11)	0.0332 (6)	
C19	0.0843 (3)	0.23988 (13)	0.75496 (12)	0.0417 (6)	
C20	−0.0926 (3)	0.22971 (15)	0.78852 (12)	0.0495 (7)	
C21	−0.1929 (3)	0.17270 (16)	0.76076 (12)	0.0503 (7)	
C22	−0.1210 (3)	0.12697 (15)	0.69684 (12)	0.0443 (7)	
C23	0.1699 (2)	0.13106 (13)	0.51121 (11)	0.0310 (6)	
C24	0.2456 (2)	0.04932 (12)	0.47505 (10)	0.0284 (5)	
C25	0.2384 (2)	−0.03413 (12)	0.54576 (11)	0.0303 (5)	
C26	0.2990 (3)	−0.13494 (12)	0.54268 (12)	0.0379 (6)	
C27	0.3760 (2)	0.13371 (12)	0.25320 (10)	0.0263 (5)	
C28	0.3623 (2)	0.22144 (12)	0.19267 (11)	0.0306 (5)	
C29	0.4246 (2)	0.22550 (12)	0.10800 (11)	0.0324 (6)	
C30	0.4999 (2)	0.14203 (12)	0.08492 (11)	0.0306 (6)	
C31	0.5177 (2)	0.05511 (12)	0.14479 (11)	0.0316 (6)	
C32	0.4578 (2)	0.05063 (12)	0.22955 (11)	0.0302 (6)	
H2	0.15770	0.84740	0.77700	0.0570*	
H3	0.02820	0.97620	0.83220	0.0900*	
H4	−0.10380	0.95290	0.97120	0.1100*	
H5	−0.11540	0.80060	1.05550	0.0930*	
H7	0.135 (2)	0.6333 (13)	0.6166 (11)	0.040 (5)*	
H10A	0.53790	0.44200	0.93550	0.0690*	
H10B	0.60330	0.44670	0.83620	0.0690*	
H10C	0.43690	0.39110	0.87740	0.0690*	
H12	0.08310	0.65070	0.46640	0.0410*	
H13	0.11360	0.58480	0.34520	0.0440*	
H15	0.41360	0.34340	0.49060	0.0420*	
H16	0.38160	0.40760	0.61240	0.0390*	
H8	0.252 (2)	0.1836 (13)	0.3497 (12)	0.040 (5)*	
H19	0.15420	0.27900	0.77470	0.0500*	
H20	−0.14490	0.26260	0.83110	0.0590*	
H21	−0.31260	0.16450	0.78560	0.0600*	
H22	−0.19250	0.08870	0.67670	0.0530*	
H26A	0.27280	−0.17660	0.59930	0.0570*	
H26B	0.43350	−0.14620	0.53010	0.0570*	
H26C	0.23080	−0.14830	0.49650	0.0570*	
H28	0.31030	0.27800	0.20970	0.0370*	
H29	0.41610	0.28480	0.06590	0.0390*	
H31	0.57090	−0.00110	0.12750	0.0380*	
H32	0.47180	−0.00860	0.27180	0.0360*	

### Atomic displacement parameters (Å^2^)

**Table:**

	*U*^11^	*U*^22^	*U*^33^	*U*^12^	*U*^13^	*U*^23^
Cl1	0.0652 (4)	0.1022 (5)	0.0431 (3)	0.0082 (3)	0.0134 (3)	0.0083 (3)
O1	0.0404 (7)	0.0362 (7)	0.0283 (7)	−0.0009 (6)	−0.0055 (6)	−0.0072 (6)
O2	0.0640 (10)	0.0469 (9)	0.0502 (9)	−0.0167 (7)	0.0170 (7)	−0.0278 (7)
O3	0.0732 (10)	0.0675 (10)	0.0328 (8)	−0.0217 (8)	−0.0005 (7)	−0.0177 (7)
N1	0.0385 (9)	0.0318 (8)	0.0303 (8)	0.0003 (7)	−0.0039 (7)	−0.0042 (7)
N2	0.0385 (8)	0.0304 (8)	0.0274 (8)	0.0003 (6)	−0.0035 (7)	−0.0087 (6)
N3	0.0362 (8)	0.0349 (8)	0.0302 (8)	−0.0073 (7)	0.0034 (7)	−0.0083 (7)
N4	0.0374 (9)	0.0304 (9)	0.0294 (8)	−0.0020 (7)	0.0016 (7)	−0.0101 (7)
N5	0.0531 (11)	0.0469 (10)	0.0357 (10)	−0.0223 (9)	0.0117 (8)	−0.0168 (8)
C1	0.0342 (10)	0.0395 (11)	0.0364 (11)	0.0042 (8)	−0.0118 (8)	−0.0180 (9)
C2	0.0425 (12)	0.0357 (11)	0.0646 (14)	0.0002 (9)	−0.0204 (10)	−0.0152 (10)
C3	0.0645 (17)	0.0444 (14)	0.122 (2)	0.0115 (12)	−0.0504 (17)	−0.0375 (16)
C4	0.0711 (19)	0.093 (2)	0.129 (3)	0.0405 (16)	−0.0581 (19)	−0.085 (2)
C5	0.0541 (15)	0.123 (2)	0.0606 (16)	0.0365 (16)	−0.0269 (12)	−0.0621 (18)
C6	0.0436 (12)	0.0706 (15)	0.0355 (11)	0.0138 (10)	−0.0139 (9)	−0.0212 (11)
C7	0.0329 (10)	0.0316 (10)	0.0269 (9)	−0.0078 (8)	0.0020 (8)	−0.0071 (8)
C8	0.0337 (10)	0.0292 (9)	0.0286 (10)	−0.0057 (8)	0.0036 (7)	−0.0087 (8)
C9	0.0360 (10)	0.0316 (10)	0.0300 (10)	−0.0042 (8)	0.0028 (8)	−0.0046 (8)
C10	0.0519 (12)	0.0399 (11)	0.0392 (11)	0.0053 (9)	−0.0015 (9)	−0.0055 (9)
C11	0.0322 (10)	0.0320 (10)	0.0304 (10)	−0.0096 (8)	0.0061 (7)	−0.0122 (8)
C12	0.0350 (10)	0.0322 (10)	0.0359 (10)	−0.0047 (8)	0.0009 (8)	−0.0084 (8)
C13	0.0402 (11)	0.0401 (11)	0.0293 (10)	−0.0107 (9)	0.0024 (8)	−0.0072 (8)
C14	0.0390 (10)	0.0369 (10)	0.0307 (10)	−0.0154 (8)	0.0086 (8)	−0.0143 (8)
C15	0.0382 (10)	0.0291 (10)	0.0376 (11)	−0.0070 (8)	0.0085 (8)	−0.0107 (8)
C16	0.0364 (10)	0.0314 (10)	0.0303 (10)	−0.0061 (8)	0.0026 (8)	−0.0072 (8)
Cl2	0.0379 (3)	0.0390 (3)	0.0408 (3)	−0.0072 (2)	−0.0009 (2)	−0.0045 (2)
O4	0.0464 (8)	0.0364 (7)	0.0264 (7)	−0.0047 (6)	0.0047 (6)	−0.0062 (6)
O5	0.0758 (10)	0.0461 (8)	0.0401 (8)	−0.0067 (7)	0.0197 (7)	−0.0230 (7)
O6	0.1420 (16)	0.0506 (10)	0.0322 (9)	0.0191 (10)	0.0312 (9)	0.0043 (8)
N6	0.0407 (9)	0.0369 (9)	0.0240 (8)	−0.0084 (7)	0.0068 (7)	−0.0078 (7)
N7	0.0346 (8)	0.0387 (9)	0.0279 (8)	−0.0092 (7)	0.0018 (6)	−0.0063 (7)
N8	0.0271 (8)	0.0363 (8)	0.0240 (8)	−0.0066 (6)	−0.0010 (6)	−0.0068 (7)
N9	0.0341 (8)	0.0292 (8)	0.0238 (8)	−0.0014 (7)	0.0032 (6)	−0.0084 (7)
N10	0.0516 (10)	0.0410 (10)	0.0314 (9)	−0.0039 (8)	0.0125 (8)	−0.0125 (8)
C17	0.0376 (10)	0.0413 (11)	0.0204 (9)	−0.0029 (8)	0.0022 (7)	−0.0067 (8)
C18	0.0359 (10)	0.0345 (10)	0.0241 (9)	0.0012 (8)	−0.0012 (8)	−0.0028 (8)
C19	0.0504 (12)	0.0427 (11)	0.0298 (10)	0.0011 (9)	−0.0070 (9)	−0.0099 (9)
C20	0.0506 (13)	0.0653 (14)	0.0271 (11)	0.0139 (11)	−0.0031 (9)	−0.0173 (10)
C21	0.0360 (11)	0.0856 (16)	0.0260 (10)	−0.0011 (11)	0.0032 (8)	−0.0151 (11)
C22	0.0363 (11)	0.0712 (14)	0.0270 (10)	−0.0124 (10)	0.0019 (8)	−0.0134 (10)
C23	0.0306 (9)	0.0399 (11)	0.0225 (9)	−0.0065 (8)	0.0011 (7)	−0.0078 (8)
C24	0.0256 (9)	0.0367 (10)	0.0234 (9)	−0.0060 (7)	0.0007 (7)	−0.0083 (8)
C25	0.0267 (9)	0.0401 (10)	0.0253 (9)	−0.0106 (8)	−0.0007 (7)	−0.0067 (8)
C26	0.0420 (11)	0.0385 (11)	0.0329 (10)	−0.0102 (9)	0.0015 (8)	−0.0063 (9)
C27	0.0256 (9)	0.0338 (9)	0.0213 (9)	−0.0044 (7)	0.0009 (7)	−0.0107 (7)
C28	0.0361 (10)	0.0284 (9)	0.0273 (9)	−0.0013 (8)	0.0018 (8)	−0.0104 (8)
C29	0.0388 (10)	0.0298 (10)	0.0259 (9)	−0.0026 (8)	0.0034 (8)	−0.0050 (8)
C30	0.0347 (10)	0.0344 (10)	0.0234 (9)	−0.0057 (8)	0.0051 (7)	−0.0098 (8)
C31	0.0362 (10)	0.0293 (10)	0.0312 (10)	−0.0043 (8)	0.0060 (8)	−0.0133 (8)
C32	0.0344 (10)	0.0265 (9)	0.0286 (10)	−0.0044 (7)	0.0020 (7)	−0.0057 (8)

### Geometric parameters (Å, º)

**Table:**

Cl1—C6	1.721 (2)	C14—C15	1.380 (2)
Cl2—C18	1.7367 (16)	C15—C16	1.374 (2)
O1—C7	1.235 (2)	C2—H2	0.9500
O2—N5	1.233 (2)	C3—H3	0.9500
O3—N5	1.235 (2)	C4—H4	0.9500
O4—C23	1.232 (2)	C5—H5	0.9500
O5—N10	1.219 (2)	C10—H10A	0.9800
O6—N10	1.221 (2)	C10—H10B	0.9800
N1—C9	1.295 (2)	C10—H10C	0.9800
N1—N2	1.426 (2)	C12—H12	0.9500
N2—C1	1.417 (2)	C13—H13	0.9500
N2—C7	1.360 (2)	C15—H15	0.9500
N3—C8	1.302 (2)	C16—H16	0.9500
N3—N4	1.334 (2)	C17—C22	1.386 (3)
N4—C11	1.390 (2)	C17—C18	1.385 (2)
N5—C14	1.461 (2)	C18—C19	1.383 (3)
N4—H7	0.910 (19)	C19—C20	1.383 (3)
N6—C23	1.371 (2)	C20—C21	1.368 (3)
N6—N7	1.423 (2)	C21—C22	1.381 (3)
N6—C17	1.417 (2)	C23—C24	1.471 (3)
N7—C25	1.300 (2)	C24—C25	1.446 (2)
N8—N9	1.317 (2)	C25—C26	1.489 (3)
N8—C24	1.310 (2)	C27—C32	1.396 (2)
N9—C27	1.400 (2)	C27—C28	1.393 (2)
N10—C30	1.460 (2)	C28—C29	1.376 (2)
N9—H8	0.863 (19)	C29—C30	1.385 (3)
C1—C6	1.385 (3)	C30—C31	1.378 (2)
C1—C2	1.384 (3)	C31—C32	1.372 (2)
C2—C3	1.371 (3)	C19—H19	0.9500
C3—C4	1.370 (5)	C20—H20	0.9500
C4—C5	1.374 (5)	C21—H21	0.9500
C5—C6	1.392 (4)	C22—H22	0.9500
C7—C8	1.472 (2)	C26—H26A	0.9800
C8—C9	1.443 (2)	C26—H26B	0.9800
C9—C10	1.490 (3)	C26—H26C	0.9800
C11—C16	1.393 (2)	C28—H28	0.9500
C11—C12	1.394 (2)	C29—H29	0.9500
C12—C13	1.378 (3)	C31—H31	0.9500
C13—C14	1.381 (3)	C32—H32	0.9500
			
Cl1···N1	3.0568 (16)	C12···C14^iii^	3.557 (2)
Cl1···N2	3.0352 (15)	C13···C12^iii^	3.557 (2)
Cl2···O4	3.3366 (12)	C13···C11^iii^	3.316 (2)
Cl2···N6	2.9917 (16)	C14···C12^iii^	3.557 (2)
Cl2···C15	3.4770 (18)	C15···O4	3.417 (2)
Cl2···C16	3.5002 (19)	C15···Cl2	3.4770 (18)
Cl2···C21^i^	3.481 (2)	C15···C11^iv^	3.348 (2)
Cl2···C22^i^	3.604 (2)	C15···C16^iv^	3.468 (2)
Cl2···C23	3.2713 (18)	C16···C16^iv^	3.451 (2)
Cl2···H15	2.9300	C16···Cl2	3.5002 (19)
Cl2···H16	2.9700	C16···C15^iv^	3.468 (2)
Cl2···H21^i^	2.9800	C18···O4	3.355 (2)
Cl2···H32^ii^	2.8000	C20···C5^xiv^	3.468 (3)
O1···C2	3.108 (2)	C21···Cl2^xvi^	3.481 (2)
O1···O3^iii^	3.2371 (19)	C22···Cl2^xvi^	3.604 (2)
O1···N3	3.0341 (19)	C23···C26^xii^	3.545 (3)
O1···N4	2.777 (2)	C23···Cl2	3.2713 (18)
O1···C28^iii^	3.396 (2)	C25···N8^ii^	3.407 (2)
O1···O4^iii^	3.1049 (16)	C26···C23^xii^	3.545 (3)
O1···N9^iii^	3.094 (2)	C28···O1^iii^	3.396 (2)
O2···N9	3.266 (2)	C28···C1^iii^	3.516 (2)
O2···C8^iv^	3.189 (2)	C28···O2	3.265 (2)
O2···C28	3.265 (2)	C29···C1^iv^	3.594 (2)
O2···O4	3.1526 (18)	C29···C2^iv^	3.514 (3)
O2···N3^iv^	3.188 (2)	C29···C6^iii^	3.341 (3)
O3···C10^iv^	3.392 (3)	C29···C5^iii^	3.391 (3)
O3···O1^iii^	3.2371 (19)	C30···C3^iv^	3.481 (3)
O3···C7^iii^	3.362 (2)	C30···C2^iv^	3.322 (3)
O4···C15	3.417 (2)	C30···C5^iii^	3.407 (3)
O4···O1^iii^	3.1049 (16)	C30···C4^iii^	3.502 (3)
O4···Cl2	3.3366 (12)	C31···O5^v^	3.343 (2)
O4···N8	3.077 (2)	C31···C3^iv^	3.421 (3)
O4···O2	3.1526 (18)	C31···C2^iv^	3.497 (3)
O4···C18	3.355 (2)	C5···H20^xiv^	3.0000
O4···N9	2.8647 (19)	C7···H7	2.476 (17)
O5···C31^v^	3.343 (2)	C7···H2	2.8400
O5···C4^vi^	3.202 (3)	C19···H13^iii^	2.8500
O5···O5^v^	3.102 (2)	C19···H5^xiv^	2.9100
O6···C10^vii^	3.175 (3)	C20···H13^iii^	2.9900
O1···H7	2.084 (18)	C20···H5^xiv^	2.8500
O1···H28^iii^	2.8500	C21···H3^xi^	3.0300
O1···H26A^viii^	2.7200	C22···H3^xi^	2.7700
O1···H8^iii^	2.478 (17)	C23···H8	2.539 (18)
O1···H2	2.7400	C23···H26C^xii^	2.8900
O2···H15	2.4200	C23···H26B^ii^	2.9600
O2···H8	2.654 (18)	C24···H26B^ii^	2.9300
O2···H28	2.5700	H2···O1	2.7400
O3···H10B^iv^	2.5200	H2···N7^viii^	2.8500
O3···H13	2.4500	H2···C7	2.8400
O4···H12^iii^	2.6500	H3···C21^viii^	3.0300
O4···H8	2.222 (18)	H3···C22^viii^	2.7700
O4···H7^iii^	2.876 (17)	H4···O5^xiii^	2.3700
O5···H4^vi^	2.3700	H4···H4^xvii^	2.5600
O5···H31	2.4300	H5···C19^xiv^	2.9100
O5···H31^v^	2.7700	H5···C20^xiv^	2.8500
O6···H10C^vii^	2.4300	H5···H20^xiv^	2.5600
O6···H20^ix^	2.9100	H7···O1	2.084 (18)
O6···H21^ix^	2.8900	H7···O4^iii^	2.876 (17)
O6···H29	2.4400	H7···C7	2.476 (17)
N1···Cl1	3.0568 (16)	H7···H12	2.3600
N2···Cl1	3.0352 (15)	H8···O4	2.222 (18)
N3···O2^iv^	3.188 (2)	H8···O1^iii^	2.478 (17)
N3···O1	3.0341 (19)	H8···C23	2.539 (18)
N4···O1	2.777 (2)	H8···H28	2.3600
N6···Cl2	2.9917 (16)	H8···O2	2.654 (18)
N8···C25^ii^	3.407 (2)	H10A···N1^x^	2.8400
N8···O4	3.077 (2)	H10B···O3^iv^	2.5200
N9···O1^iii^	3.094 (2)	H10C···O6^xv^	2.4300
N9···O2	3.266 (2)	H12···H7	2.3600
N9···O4	2.8647 (19)	H12···O4^iii^	2.6500
N1···H10A^x^	2.8400	H13···O3	2.4500
N3···H16	2.4700	H13···C19^iii^	2.8500
N7···H2^xi^	2.8500	H13···C20^iii^	2.9900
N8···H22^xii^	2.8400	H15···Cl2	2.9300
N8···H32	2.4800	H15···O2	2.4200
C1···C28^iii^	3.516 (2)	H16···Cl2	2.9700
C1···C29^iv^	3.594 (2)	H16···N3	2.4700
C2···O1	3.108 (2)	H20···O6^xviii^	2.9100
C2···C29^iv^	3.514 (3)	H20···C5^xiv^	3.0000
C2···C30^iv^	3.322 (3)	H20···H5^xiv^	2.5600
C2···C31^iv^	3.497 (3)	H21···Cl2^xvi^	2.9800
C3···C31^iv^	3.421 (3)	H21···O6^xviii^	2.8900
C3···C30^iv^	3.481 (3)	H22···N8^xii^	2.8400
C4···O5^xiii^	3.202 (3)	H22···H32^xii^	2.5300
C4···C30^iii^	3.502 (3)	H26A···O1^xi^	2.7200
C5···C29^iii^	3.391 (3)	H26B···C23^ii^	2.9600
C5···C20^xiv^	3.468 (3)	H26B···C24^ii^	2.9300
C5···C30^iii^	3.407 (3)	H26C···C23^xii^	2.8900
C6···C29^iii^	3.341 (3)	H28···O2	2.5700
C7···O3^iii^	3.362 (2)	H28···H8	2.3600
C8···O2^iv^	3.189 (2)	H28···O1^iii^	2.8500
C10···O6^xv^	3.175 (3)	H29···O6	2.4400
C10···O3^iv^	3.392 (3)	H31···O5	2.4300
C11···C15^iv^	3.348 (2)	H31···O5^v^	2.7700
C11···C13^iii^	3.316 (2)	H32···N8	2.4800
C12···C13^iii^	3.557 (2)	H32···Cl2^ii^	2.8000
C12···C12^iii^	3.594 (2)	H32···H22^xii^	2.5300
			
N2—N1—C9	106.83 (13)	H10A—C10—H10B	109.00
N1—N2—C1	119.93 (13)	H10A—C10—H10C	109.00
N1—N2—C7	112.75 (14)	C9—C10—H10A	110.00
C1—N2—C7	127.10 (15)	C11—C12—H12	120.00
N4—N3—C8	117.15 (15)	C13—C12—H12	120.00
N3—N4—C11	119.70 (15)	C14—C13—H13	120.00
O2—N5—O3	123.58 (17)	C12—C13—H13	120.00
O2—N5—C14	118.30 (15)	C14—C15—H15	120.00
O3—N5—C14	118.12 (16)	C16—C15—H15	120.00
C11—N4—H7	118.9 (11)	C11—C16—H16	120.00
N3—N4—H7	121.4 (11)	C15—C16—H16	120.00
C17—N6—C23	126.83 (15)	C18—C17—C22	119.34 (16)
N7—N6—C23	112.99 (14)	N6—C17—C22	119.98 (16)
N7—N6—C17	120.19 (13)	N6—C17—C18	120.68 (14)
N6—N7—C25	106.64 (13)	Cl2—C18—C17	120.02 (13)
N9—N8—C24	118.23 (15)	Cl2—C18—C19	119.47 (14)
N8—N9—C27	119.93 (15)	C17—C18—C19	120.47 (16)
O5—N10—O6	122.44 (16)	C18—C19—C20	119.38 (18)
O5—N10—C30	118.93 (15)	C19—C20—C21	120.46 (19)
O6—N10—C30	118.63 (16)	C20—C21—C22	120.3 (2)
N8—N9—H8	121.6 (12)	C17—C22—C21	120.0 (2)
C27—N9—H8	118.3 (12)	O4—C23—N6	127.09 (17)
C2—C1—C6	119.81 (19)	O4—C23—C24	129.70 (15)
N2—C1—C2	118.81 (16)	N6—C23—C24	103.21 (15)
N2—C1—C6	121.35 (18)	N8—C24—C23	128.65 (16)
C1—C2—C3	120.1 (2)	C23—C24—C25	106.06 (14)
C2—C3—C4	120.3 (3)	N8—C24—C25	125.28 (16)
C3—C4—C5	120.5 (3)	N7—C25—C26	121.74 (16)
C4—C5—C6	119.7 (3)	C24—C25—C26	127.17 (15)
C1—C6—C5	119.6 (2)	N7—C25—C24	111.09 (15)
Cl1—C6—C5	118.78 (17)	C28—C27—C32	120.65 (15)
Cl1—C6—C1	121.59 (17)	N9—C27—C28	118.40 (16)
O1—C7—C8	128.04 (16)	N9—C27—C32	120.95 (15)
O1—C7—N2	128.49 (16)	C27—C28—C29	119.44 (16)
N2—C7—C8	103.47 (14)	C28—C29—C30	119.11 (16)
C7—C8—C9	105.96 (14)	N10—C30—C31	119.01 (16)
N3—C8—C7	127.95 (15)	N10—C30—C29	119.11 (15)
N3—C8—C9	126.04 (15)	C29—C30—C31	121.87 (16)
N1—C9—C10	121.84 (15)	C30—C31—C32	119.33 (16)
C8—C9—C10	127.23 (16)	C27—C32—C31	119.53 (16)
N1—C9—C8	110.92 (15)	C18—C19—H19	120.00
N4—C11—C16	120.98 (15)	C20—C19—H19	120.00
C12—C11—C16	120.35 (16)	C19—C20—H20	120.00
N4—C11—C12	118.67 (16)	C21—C20—H20	120.00
C11—C12—C13	119.63 (16)	C20—C21—H21	120.00
C12—C13—C14	119.22 (16)	C22—C21—H21	120.00
N5—C14—C15	118.64 (16)	C17—C22—H22	120.00
C13—C14—C15	121.69 (16)	C21—C22—H22	120.00
N5—C14—C13	119.66 (15)	C25—C26—H26A	109.00
C14—C15—C16	119.39 (16)	C25—C26—H26B	109.00
C11—C16—C15	119.71 (16)	C25—C26—H26C	109.00
C1—C2—H2	120.00	H26A—C26—H26B	109.00
C3—C2—H2	120.00	H26A—C26—H26C	110.00
C4—C3—H3	120.00	H26B—C26—H26C	109.00
C2—C3—H3	120.00	C27—C28—H28	120.00
C5—C4—H4	120.00	C29—C28—H28	120.00
C3—C4—H4	120.00	C28—C29—H29	120.00
C4—C5—H5	120.00	C30—C29—H29	120.00
C6—C5—H5	120.00	C30—C31—H31	120.00
C9—C10—H10C	109.00	C32—C31—H31	120.00
C9—C10—H10B	109.00	C27—C32—H32	120.00
H10B—C10—H10C	110.00	C31—C32—H32	120.00
			
C9—N1—N2—C1	−177.60 (14)	C3—C4—C5—C6	−0.1 (4)
C9—N1—N2—C7	−2.68 (18)	C4—C5—C6—Cl1	−175.3 (2)
N2—N1—C9—C10	−179.42 (15)	C4—C5—C6—C1	1.6 (3)
N2—N1—C9—C8	1.49 (18)	N2—C7—C8—C9	−1.58 (16)
C7—N2—C1—C2	−45.7 (2)	N2—C7—C8—N3	−178.96 (16)
N1—N2—C1—C6	−53.3 (2)	O1—C7—C8—N3	1.4 (3)
C7—N2—C1—C6	132.57 (19)	O1—C7—C8—C9	178.79 (16)
C1—N2—C7—C8	177.05 (15)	N3—C8—C9—N1	177.47 (16)
N1—N2—C7—O1	−177.80 (16)	N3—C8—C9—C10	−1.6 (3)
C1—N2—C7—O1	−3.3 (3)	C7—C8—C9—C10	−179.01 (16)
N1—N2—C1—C2	128.42 (18)	C7—C8—C9—N1	0.03 (19)
N1—N2—C7—C8	2.58 (17)	N4—C11—C12—C13	−179.02 (15)
C8—N3—N4—C11	−179.70 (15)	N4—C11—C16—C15	179.38 (15)
N4—N3—C8—C7	1.0 (2)	C12—C11—C16—C15	−0.3 (2)
N4—N3—C8—C9	−175.90 (15)	C16—C11—C12—C13	0.7 (2)
N3—N4—C11—C16	6.4 (2)	C11—C12—C13—C14	−0.4 (2)
N3—N4—C11—C12	−173.89 (15)	C12—C13—C14—N5	178.73 (14)
O2—N5—C14—C15	−10.7 (2)	C12—C13—C14—C15	−0.2 (2)
O3—N5—C14—C13	−10.0 (2)	C13—C14—C15—C16	0.5 (2)
O2—N5—C14—C13	170.37 (15)	N5—C14—C15—C16	−178.37 (14)
O3—N5—C14—C15	168.98 (15)	C14—C15—C16—C11	−0.3 (2)
N7—N6—C17—C22	−65.3 (2)	C22—C17—C18—Cl2	175.76 (14)
C23—N6—C17—C18	−66.0 (2)	N6—C17—C18—Cl2	−3.7 (2)
C23—N6—C17—C22	114.6 (2)	C22—C17—C18—C19	−1.8 (3)
C17—N6—C23—C24	178.77 (15)	N6—C17—C18—C19	178.76 (16)
N7—N6—C17—C18	114.09 (18)	C18—C17—C22—C21	0.4 (3)
N7—N6—C23—O4	178.25 (15)	N6—C17—C22—C21	179.79 (18)
C17—N6—C23—O4	−1.7 (3)	C17—C18—C19—C20	1.3 (3)
N7—N6—C23—C24	−1.28 (17)	Cl2—C18—C19—C20	−176.25 (15)
C23—N6—N7—C25	1.31 (18)	C18—C19—C20—C21	0.6 (3)
C17—N6—N7—C25	−178.74 (14)	C19—C20—C21—C22	−2.1 (3)
N6—N7—C25—C24	−0.71 (17)	C20—C21—C22—C17	1.6 (3)
N6—N7—C25—C26	179.30 (15)	O4—C23—C24—N8	2.4 (3)
C24—N8—N9—C27	179.13 (14)	O4—C23—C24—C25	−178.71 (16)
N9—N8—C24—C25	179.09 (14)	N6—C23—C24—N8	−178.13 (16)
N9—N8—C24—C23	−2.2 (2)	N6—C23—C24—C25	0.80 (16)
N8—N9—C27—C28	177.73 (14)	N8—C24—C25—N7	178.93 (15)
N8—N9—C27—C32	−2.2 (2)	N8—C24—C25—C26	−1.1 (3)
O5—N10—C30—C31	−5.4 (2)	C23—C24—C25—N7	−0.05 (18)
O5—N10—C30—C29	175.80 (15)	C23—C24—C25—C26	179.94 (16)
O6—N10—C30—C29	−4.4 (2)	N9—C27—C28—C29	−177.71 (14)
O6—N10—C30—C31	174.38 (17)	C32—C27—C28—C29	2.2 (2)
N2—C1—C6—C5	179.79 (18)	N9—C27—C32—C31	176.99 (14)
C2—C1—C6—Cl1	174.84 (16)	C28—C27—C32—C31	−2.9 (2)
C6—C1—C2—C3	0.8 (3)	C27—C28—C29—C30	−0.1 (2)
C2—C1—C6—C5	−2.0 (3)	C28—C29—C30—N10	177.47 (14)
N2—C1—C6—Cl1	−3.4 (3)	C28—C29—C30—C31	−1.3 (2)
N2—C1—C2—C3	179.09 (19)	N10—C30—C31—C32	−178.19 (14)
C1—C2—C3—C4	0.7 (4)	C29—C30—C31—C32	0.6 (2)
C2—C3—C4—C5	−1.1 (4)	C30—C31—C32—C27	1.5 (2)

Symmetry codes: (i) *x*+1, *y*, *z*; (ii) −*x*+1, −*y*, −*z*+1; (iii) −*x*, −*y*+1, −*z*+1; (iv) −*x*+1, −*y*+1, −*z*+1; (v) −*x*+1, −*y*, −*z*; (vi) *x*+1, *y*−1, *z*−1; (vii) *x*, *y*, *z*−1; (viii) *x*, *y*+1, *z*; (ix) *x*+1, *y*, *z*−1; (x) −*x*+1, −*y*+1, −*z*+2; (xi) *x*, *y*−1, *z*; (xii) −*x*, −*y*, −*z*+1; (xiii) *x*−1, *y*+1, *z*+1; (xiv) −*x*, −*y*+1, −*z*+2; (xv) *x*, *y*, *z*+1; (xvi) *x*−1, *y*, *z*; (xvii) −*x*, −*y*+2, −*z*+2; (xviii) *x*−1, *y*, *z*+1.

### Hydrogen-bond geometry (Å, º)

**Table:**

*D*—H···*A*	*D*—H	H···*A*	*D*···*A*	*D*—H···*A*
N4—H7···O1	0.910 (19)	2.084 (18)	2.777 (2)	132.1 (14)
N9—H8···O4	0.863 (19)	2.222 (18)	2.8647 (19)	131.2 (16)
C28—H28···O2	0.95	2.57	3.265 (2)	130
C15—H15···Cl2	0.95	2.93	3.4770 (18)	118
N9—H8···O2	0.86 (2)	2.654 (18)	3.266 (2)	129.0 (13)
N9—H8···O1^iii^	0.863 (19)	2.478 (17)	3.094 (2)	128.9 (14)
C4—H4···O5^xiii^	0.95	2.37	3.202 (3)	146
C10—H10*B*···O3^iv^	0.98	2.52	3.392 (3)	148
C10—H10*C*···O6^xv^	0.98	2.43	3.175 (3)	133
C32—H32···Cl2^ii^	0.95	2.80	3.6898 (18)	157

Symmetry codes: (ii) −*x*+1, −*y*, −*z*+1; (iii) −*x*, −*y*+1, −*z*+1; (iv) −*x*+1, −*y*+1, −*z*+1; (xiii) *x*−1, *y*+1, *z*+1; (xv) *x*, *y*, *z*+1.
